# Gene model for the ortholog of *Cyp6a14* in *Drosophila cardini*

**DOI:** 10.17912/micropub.biology.002297

**Published:** 2026-07-30

**Authors:** Stephen Moxley, Pablo Chialvo, Clare Scott Chialvo

**Affiliations:** 1 Chemistry, Appalachian State University, Boone, North Carolina USA; 2 Biology, Appalachian State University, Boone, North Carolina USA

## Abstract

We developed a gene model for the *Cytochrome P450 6a14* ortholog ( *Cyp6a14* ) in the ASM1890373v1 Genome Assembly (GenBank Accession: GCA_018903735.1 ) of *Drosophila cardini* . This ortholog was characterized as part of a developing dataset for a comparative study of detoxification gene family evolution in the *immigrans* - *tripunctata* radiation of the genus *Drosophila* using an adapted Genomics Education Partnership gene annotation protocol for Course-based Undergraduate Research Experiences.

**Figure 1. Genomic neighborhood and gene model for Cyp6a14 in D. cardini:** (A) **Synteny comparison of the genomic neighborhoods for *Cyp6a14* in *Drosophila melanogaster* and *D. cardini* .** Thin underlying arrows indicate which DNA strand the target gene– *Cyp6a14* –is located on in *D. melanogaster* (top) and *D. cardini* (bottom). The thin arrows pointing to the left indicate that *Cyp6a14* is on the negative strand in both *D. melanogaster* and *D. cardini* . The wide gene arrows pointing in the same direction as *Cyp6a14* are on the same strand relative to the thin underlying arrows, while wide gene arrows pointing in the opposite direction of *Cyp6a14* are on the opposite strand relative to the thin underlying arrows. White gene arrows in *D. cardini* indicate orthology to the corresponding gene in *D. melanogaster* , gray arrows indicate that the gene is present in both genomic neighborhoods but not syntenic ( *Cyp6a13* , *mtt* , *Mal-A8* ), and black gene arrows indicate non-orthology. Gene symbols given in the *D. cardini* gene arrows indicate the orthologous gene in *D. melanogaster* , while the locus identifiers are specific to *D. cardini* . (B) **Gene Model in GEP UCSC Track Data Hub** (Raney et al., 2014). The coding-regions of *Cyp6a14* in *D. cardini* are displayed in the User Supplied Track (red); coding sequences (CDS) are depicted by thick rectangles and introns by thin lines with arrows indicating the direction of transcription. Subsequent evidence tracks include Spaln of D. melanogaster Proteins (purple, alignment of Ref-Seq proteins from *D. melanogaster* ), Coding Regions Predicted by Augustus (dark blue), GeMoMa (teal), and NSCAN PASA-EST (dark green), GlimmerHMM (gray), and RNA-Seq from mixed sex adult flies (brown; alignment of Illumina RNA-Seq reads from *D. cardini* – Erlenbach et al. 2023). (C) **Dot Plot of Cyp6a14-PE in *D. melanogaster* ( *x* -axis) vs. the orthologous peptide in *D. cardini* ( *y* -axis).** Amino acid number is indicated along the left and bottom; CDS number is indicated along the top and right, and CDSs are also highlighted with alternating colors. Line breaks in the dot plot indicate areas of low sequence identity between species. At the beginning of CDS 1, there is an area of approximately 180 amino acids (dark purple box – a) that has four short areas of sequence similarity. There is also a short break at the end of this CDS (light blue box – b). In CDS 2, there are two short breaks. One is at the beginning of the CDS (dark green box – c). The other is at the end of the CDS (yellow box – d). (D) **Idiosyncrasies in protein alignment.** We noted four areas in the protein alignments that indicate low levels of sequence similarity. Two of these areas are found in CDS 1 and the others are in CDS 2. The first area in CDS 1 (dark purple box – a) extends over the first 180 amino acids, but only 18 of these amino acids are highly dissimilar. The second area in CDS 1 is a short break (light blue box – b) that spans 24 amino acids (21 of these are similar and only one is highly dissimilar. The short break at the beginning of CDS 2 (dark green box – c) covers 29 amino acids. Of these, only five of the amino acids are highly dissimilar and 21 are similar. The fourth break (yellow box – d) is towards the end of CDS 2 and spans 22 amino acids and only 2 of them are dissimilar.

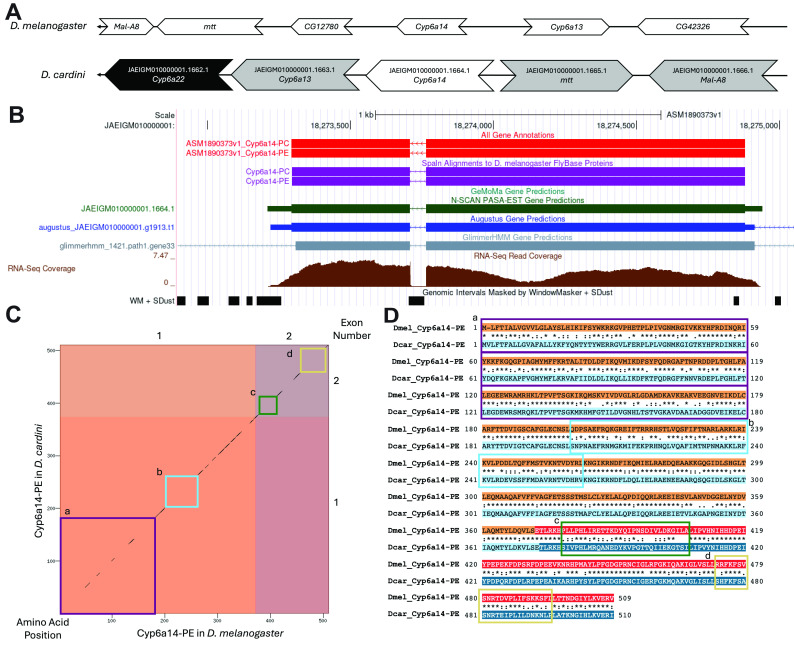



## Description

**Table:** 

*This article reports a predicted gene model generated by undergraduate work using a structured gene model annotation protocol defined by the Genomics Education Partnership (GEP; thegep.org) for Course-based Undergraduate Research Experience (CURE). The following information in quotes may be repeated in other articles submitted by participants using the same GEP CURE protocol for annotating Drosophila species orthologs of Drosophila melanogaster detoxification genes.* ** ** ***“*** Within insects, the process of detoxifying xenobiotics and host secondary metabolites is a three-phase process that involves functionalization, conjugation, and excretion of these compounds. Expansions of known detoxification gene families ( *e.g.* , cytochrome P450s) is associated with diet breadth and insecticide resistance (Ranson et al., 2002; Després et al., 2007; Rane et al., 2016). With the increasing availability of high-quality genomes for non-model organisms, including *Drosophila* species beyond *D. melanogaster* , it is now possible to perform large scale comparative studies (Robinson et al., 2011; Kim et al., 2021; Threfall and Baxter 2021). Careful manual annotation and curation of gene models can improve upon computational gene predictions in non-model species, which aids the accuracy of studies on gene and genome evolution (Mudge and Harrow 2016; Tello-Ruiz et al., 2019). To aid in these annotations, the Genomics Education Partnership (thegep.org) developed a curriculum involving web-based tools that allow undergraduates to engage in authentic course-based research focused on manually annotating genes in non-model species (Rele et al., 2023). The orthologous gene models, including the one presented here, then provide a reliable basis for further evolutionary genomic analyses when made available to the scientific community. The gene ortholog described here in *D. cardini* for *Cytochrome P450 6a14* ( *Cyp6a14* ), a member of the cytochrome P450 monooxygenases gene family, was characterized as part of a developing dataset for a comparative study of detoxification gene families in the *immigrans* - *tripunctata* radiation of the genus *Drosophila* .” (Williams et al., 2026) “In the subgenus *Drosophila* , *D. cardini* Sturtevant 1916 is a member of the *cardini* subgroup in the *cardini* species group of the *immigrans-tripunctata* radiation (Heed & Krishnamurthy 1959; Bächli 2005). Species in the *cardini* subgroup are found in the mainland Neotropics, and the range of *D. cardini* extends from Florida to Brazil (Heed, 1962). Members of the *cardini* group primarily feed and develop on fruit and flowers (Markow & O'Grady 2008). However, *D. cardini* is also reported to feed on mushrooms and can tolerate the cyclopeptide toxin α-amanitin (Stump et al., 2011).” (Patel et al., 2026) “Cytochrome P450 monooxygenases (CYPs) are a family of phase I detoxification enzymes that are found in almost all aerobic organisms and act by oxidizing compounds to make them more polar (Stegeman and Livingstone, 1998; Li et al., 2007). The enzymes in this family vary in both their substrate specificity and the range of metabolites that they produce (Rendic and Di Carlo 1997; Scott 1999). Furthermore, the substrate specificity of CYPs can be altered by a change in a single amino acid (Lindberg and Negishi, 1989).” (Patel et al., 2026)

*Cytochrome P450 6a14* ( *Cyp6a14* ) is a member of the CYP6 family that is restricted to insects (Tijet et al., 2001). It shows increased expression the larval midgut where it could contribute to the metabolism of xenobiotics (Chung et al., 2009; Harrop et al., 2014).

We propose a gene model for the *D. cardini* ortholog of the *D. melanogaster* *Cytochrome P450 6a14* ( *Cyp6a14* ) gene. The genomic region of the ortholog corresponds to the NSCAN PASA-EST gene prediction JAEIGM010000001.1664.1 in the ASM1890373v1 Genome Assembly of *D. cardini* ( GCA_018903735.1 – Kim et al., 2021). This model is based on mixed sex adult RNA-Seq data from *D. cardini* (Erlenbach et al. 2023; https://doi.org/10.5061/dryad.hdr7sqvq2) and *Cyp6a14* in *D. melanogaster* using FlyBase release FB2024_02 ( GCA_000001215.4 ; Gramates et al., 2022; Jenkins et al., 2022; Larkin et al., 2021).

***Synteny***

The reference gene, *Cyp6a14 ,* occurs on chromosome 2R in *D. melanogaster* and is flanked upstream by *Cytochrome P450 6a13* ( *Cyp6a13* ) and *CG42326* and downstream by *CG12780* , *mangetout* ( *mtt* ), and *Maltase A8* ( *Mal-A8* ). The *tblastn* search of *D. melanogaster* Cyp6a14-PE (query) against the *D. cardini* (GenBank Accession: GCA_018903735.1 Genome Assembly (ASM1890373v1)) placed the putative ortholog of *Cyp6a14* within contig_2203 (JAEIGM010000001.1) which corresponds to the NSCAN PASA-EST gene prediction JAEIGM010000001.1664.1 (E-value: 0.0; percent identity: 65.42% as determined by *blastp* ). The putative ortholog is flanked upstream by the NSCAN PASA-EST gene predictions JAEIGM010000001.1665.1 and JAEIGM010000001.1666.1, which correspond to *mtt* and *Mal-A8* in *D. melanogaster* (E-value: 0.0 and 0.0; identity: 76.68% and 80.03%, respectively, as determined by *blastp* ; Figure 1A ; Altschul et al., 1990). The putative ortholog of *Cyp6a14* is flanked downstream by the NSCAN PASA-EST gene predictions JAEIGM010000001.1663.1 and JAEIGM010000001.1662.1, which correspond to *Cyp6a13* and *Cyp6a22* in *D. melanogaster* (E-value: 0.0 and 0.0; identity: 66.12% and 77.62%, respectively, as determined by *blastp* ). The putative ortholog assignment for *Cyp6a14* in *D. cardini* is supported by the following evidence: The *tblastn* results are of good quality, and all coding sequences (CDS) and isoforms found in *D. melanogaster* also appear to be present in *D. cardini* . While the gene predictions surrounding the *Cyp6a14* ortholog are not conserved, three of the genes ( *Cyp6a13* , *mtt* , and *Mal-A8* ) are found immediately upstream or downstream of *Cyp6a14* in *D. melanogaster* . However, their orientation is reversed. The remaining gene prediction ( *Cyp6a22* ) is not found in close proximity to *Cyp6a14* in *D. melanogaster* but does occur on the same chromosome. These differences suggest the potential for a chromosomal inversion in this region. Inversions are common within the genus *Drosophila* and play an important role in speciation (Powell, 1997; Bhutkar et al., 2008; Reis et al., 2018). We conclude that the NSCAN PASA-EST gene prediction JAEIGM010000001.1664.1 represents an ortholog of *Cyp6a14* in *D. cardini* ( Figure 1A ).

***Protein Model***

*Cyp6a14* in *D. cardini* has two CDSs within the genome sequence. The only unique protein sequence is translated from two messenger RNA isoforms that differ in their untranslated regions (Cyp6a14-RE, Cyp6a14-PC; Figure 1B ). Relative to the ortholog in *D. melanogaster* , the CDS number and protein isoform count are conserved *.* The sequence of Cyp6a14-PE in *D. cardini* has 65.5% identity (81.2% similarity) with the protein-coding isoform Cyp6a14-PE in *D. melanogaster* , as determined by *blastp* ( Figure 1C ). This level of divergence is not surprising given that *D. cardini* and *D. melanogaster* belong to two separate subgenera ( *Drosophila* and *Sophophora* respectively) that diverged approximately 45-60 MYA (Russo et al., 1995; Tamura et al. 2004; Obbard et al., 2012) *.* Coordinates of this curated gene model are archived in the CaltechDATA repository (see “Extended Data” section below).

## Methods

The annotation methods used in this project are adapted from those described in Rele et al. (2023), which includes algorithms, database versions, and citations for the complete annotation process developed for the Pathways Project. The methods for the current project are detailed in brief below with notes on significant differences between this protocol and the one described in Rele et al. (2023). The students use the GEP instance of the UCSC Genome Browser v.435 (https://gander.wustl.edu ; Kent WJ et al., 2002; Raney et al., 2024) to examine the genomic neighborhood of their reference detoxification gene in the *D. melanogaster* genome assembly (Aug. 2014; BDGP Release 6 + ISO1 MT/dm6). Students obtain the protein sequence for the *D. melanogaster* target gene for a given isoform and use a *tblastn* search of the sequence against their target *Drosophila* species genome assembly ( *D. cardini* ( GCA_018903735.1 – Kim et al., 2021)) on the NCBI BLAST server (https://blast.ncbi.nlm.nih.gov/Blast.cgi, Altschul et al., 1990) to identify the putative ortholog location. Students compare the genomic neighborhood of the putative ortholog to that of the reference gene in *D. melanogaster* . This local synteny analysis includes a minimum of two upstream and downstream genes relative to the potential ortholog. As no RefSeq protein data is available for these species, comparisons are based on gene predictions that correlate with gene expression data in the putative ortholog neighborhood. Using the multiple alignment tracks feature in the Genome Browser, students examine other sets of genomic evidence, including Spaln alignment of *D. melanogaster* proteins, multiple gene prediction tracks (e.g., GeMoMa, Augustus, NSCAN PASA-EST), and mixed sex RNA-Seq adult expression data from the target species generated by Erlenbach et al. (2023; https://doi.org/10.5061/dryad.hdr7sqvq2). Information on the genomic structure information (e.g., CDSs, intron-exon number, number of isoforms) for the reference gene in *D. melanogaster* is retrieved using Gene Record Finder (https://gander.wustl.edu/~wilson/dmelgenerecord/index.html; Rele et al *.,* 2023). To determine approximate splice sites within the target gene, a *tblastn* search using the CDSs from the *D. melanogaste* r reference gene against the putative ortholog location (10kb up- and downstream of the target gene prediction). Coordinates of the CDS(s) are refined by examining aligned RNA-Seq data, identifying canonical splice site sequences, and ensuring the maintenance of an open reading frame. Students confirm the biological validity of their target gene model using the FlySeq Gene Model Checker (https://gander2.wustl.edu/~wilson/genechecker-flyseq/), which compares the hypothesized target gene model's structure and translated sequence against the *D. melanogaster* reference gene. At least two independent models for this gene are generated. These models are reconciled by a third independent researcher to produce the final model presented here. Note: comparison of 5' and 3' UTR sequence information is not included in this GEP CURE protocol.

## Data Availability

Description: Zipped archive containing FASTA, PEP, and GFF files for the Cyp6a14 model. Resource Type: Dataset. DOI: https://doi.org/10.22002/x7m67-q8235
